# Sex and gender differences in primary care help-seeking for common somatic symptoms: a longitudinal study

**DOI:** 10.1080/02813432.2023.2191653

**Published:** 2023-03-30

**Authors:** Aranka V. Ballering, Tim C. Olde Hartman, Robert Verheij, Judith G. M. Rosmalen

**Affiliations:** a Department of Psychiatry, University of Groningen, University Medical Center of Groningen, Groningen, The Netherlands; b Department of Primary and Community Care, Radboud University Medical Center, Nijmegen, The Netherlands; c Nivel, Netherlands Institute of Health Services Research, Utrecht, The Netherlands; d Tilburg School of Social and Behavioral Sciences, Tranzo Scientific Center for Care and Welfare, Tilburg University, Tilburg, The Netherlands

**Keywords:** Gender roles, general practice, help-seeking behavior, medically unexplained symptoms, sex differences, primary health care

## Abstract

**Objective:**

Women are reported to consult general practitioners (GPs) more frequently than men. However, previous studies on sex differences in help-seeking behavior for somatic symptoms do not distinguish between sex and gender, do not account for sex differences in presented symptoms, and are frequently conducted in clinical settings, automatically excluding non-help seekers. Therefore, we aim to assess the independent associations of sex and gender with primary care help-seeking for somatic symptoms in the general population.

**Design and setting:**

Records from the longitudinal population-based Lifelines Cohort Study were linked to routine electronic health records from GPs.

**Subjects:**

Participants reporting new-onset common somatic symptoms.

**Main outcome measures:**

Associations between sex and gender, operationalized via a novel gender-index, with primary care help-seeking for somatic symptoms and differences in the strength of the association between gender and help-seeking for somatic symptoms between women and men.

**Results:**

Of 20,187 individuals with linked data, 8325 participants (67.5% female; mean age = 44.5 years [SD = 12.9]) reported at least one new-onset somatic symptom. Hereof, 255 (3.1%) consulted the GP within 6 weeks of symptom onset. Female sex was positively associated with consulting the GP (OR = 1.78; 95%CI = 1.13–2.80), whereas feminine gender was not (OR = 0.67; 95%CI = 0.39–1.16). The latter association did not differ in strength between men and women. More paid working days are negatively associated with help-seeking (OR = 0.95; 95%CI = 0.91–0.98).

**Conclusions:**

The results suggest that female sex rather than feminine gender is associated with primary care help-seeking behavior for somatic symptoms. Nevertheless, clinicians should be aware that gender-related variables, such as mean paid working days, may be associated with help-seeking behavior.

## Introduction

Previous studies have suggested that women more frequently consult their general practitioner (GP) for common somatic symptoms than men [1–5]. This may partly be explained by sex and gender differences in the occurrence of such symptoms: female participants reported more frequent, severe and persistent somatic symptoms, whereas femininity (i.e. feminine gender) was protective of the persistence of symptoms [6–8]. However, also help-seeking behavior for these symptoms might contribute to the increased consultation rates in women. To understand how help-seeking for somatic symptoms is affected by sex and gender, it is important to clearly distinguish between these latter two concepts [9]. Sex refers to biological characteristics, including but not limited to chromosomes, hormones and anatomy, of female and male bodies. In contrast, gender is a socioculturally-constructed, multidimensional concept that entails the embodiment of different roles, behaviors, identities and relationships of women and men prescribed by social norms in a given time and society [10].

The idea of increased female primary care help-seeking for common somaticsymptoms, however, is under debate. A recent systematic review that directly compared the consultation patterns in women and men experiencing headaches and lower back pain concluded that the evidence for a female preponderance in help-seeking was surprisingly weak and inconsistent [11]. Previous studies on sex and gender differences in primary care help-seeking for somatic symptoms are characterized by several methodological limitations.

First, potentially due to the absence of adequate gender measures in epidemiological studies, these could not quantify independent sex and gender differences in the frequency of help-seeking behavior for somatic symptoms [6,9,12–14]. Second, most studies focusing on help-seeking behavior in relation to somatic symptoms are conducted in clinical populations or patient registries, which is problematic as it automatically excludes people who do not seek help for their somatic symptoms [11]. Third, previous studies are largely based on self-reported measures of help-seeking, making these studies prone to recall bias. Fourth, most study designs do not correct for the type of somatic symptom and sex differences in the presentation of these [15].

We present the first large epidemiological cohort study on the association between sex, gender and help-seeking for common somatic symptoms that overcomes these problems. We linked the Lifelines general population cohort to the Nivel Primary Care Database (NPCD) [16], allowing us to assess independent sex and gender differences in symptom-specific help-seeking behavior in the general population. We examined whether sex and gender have a unique association with help-seeking behavior for common somatic symptoms and whether the association between gender and help-seeking behavior for common somatic symptoms varies between sexes.

## Methods

### Lifelines and the Nivel Primary Care Database

Lifelines is a multi-disciplinary prospective population-based cohort study examining in a unique three-generation design the health and health-related behaviors of 167,729 persons living in the North of The Netherlands. It employs a broad range of investigative procedures in assessing the biomedical, socio-demographic, behavioral, physical and psychological factors which contribute to health and disease of the general population, with a special focus on multi-morbidity and complex genetics. The Lifelines Cohort Study is approved by the Medical Ethical Committee of University Medical Center Groningen (2007/152) and participants provided written consent. Extensive information on the cohort, design considerations and recruitment procedures is provided elsewhere [17,18]. In this study, we included Lifelines data collected from 2008 up until 2018.

The NPCD encompasses electronic health records data from general practices located across the Netherlands, with diagnoses, contacts and prescription medicine coded via the International Classification of Primary Care (ICPC-2) system. Virtually all people residing in the Netherlands are listed as a patient in a GP practice. The NPCD population is a representative sample of the Dutch population. Data on GP consultations for common somatic symptoms as described in the Symptom CheckList-90 Somatization subscale (SCL-90 SOM; Table S1**)** were retrieved from electronic health records for all participants that were listed in one of the 63 NPCD GP practices located in the North of the Netherlands. This study has been approved according to the governance code of NPCD under number NZR-00319.049.

### Variables

The presence of self-reported common somatic symptoms was assessed in Lifelines surveys by the 12-item ordinal SCL-90 SOM, which has been recommended for large-scale studies and has sufficient measurement invariance over time [19,20]. Table S2 shows the definition of new-onset symptoms. The symptoms (ICPC) of the SCL-90 SOM comprise headache (N01), dizziness (N17), heart pain (K01), (lower) back pain (L02 and L03), nausea (D09), muscle pain (L18), shortness of breath (R02), chills (A02), tingling of fingers, feet and/or toes (N05), swallowing/throat problems (D21 and R21), general tiredness (A04) and heavy arms and/or legs (L09 and L14). The SCL-90 SOM subscale assesses the extent of distress or bother participants experienced during the past seven days due to these symptoms. Presence of the new-onset symptoms was assessed with Lifelines’ survey data, whereas GP consults for these symptoms were listed in the NPCD.

Participants’ sex (female or male) and age in years were derived from the municipal population registry. Participants’ self-reported highest attained educational level was categorized into ‘high’, ‘medium’ and ‘low’ [21]. Participants’ burden of somatic symptoms was measured by the SCL-90 SOM sumscore at the moment of reporting a new-onset symptom [22]. Participants’ feminine and masculine gender roles were operationalized via a recently developed, data-driven gender index, which accounts for the place-, time- and society-bound nature of gender roles [6]. In a subsample of baseline Lifelines adult participants that had no suspected intersex variation or gender-diverse gender identity, we performed LASSO logistic regression analyses that used 153 psychosocial characteristics, including dietary preferences, hobbies, time spend on household tasks or odd jobs, type of profession and personality traits, to predict participants’ municipally-registered sex. The majority of the included psychosocial variables referred to gender roles. Using the obtained estimates of the regression coefficients of the LASSO logistic regression model (AUC = 92%), an individual score regarding feminine and masculine gender roles (i.e. the gender index) was calculated for each adult participant. In other words, participants’ individual adherence to prototypical feminine and masculine psychosocial characteristics was calculated. The gender index ranges from 0% (fully masculine) to 100% (fully feminine). An index of 50% indicates androgyny, with equal levels of feminine and masculine characteristics present. Extensive information on the development and applicability of the gender index is provided elsewhere [6,7].

To define help-seeking behavior of participants, we linked data derived from Lifelines with the NPCD (for detailed procedure: Figure S1). A Dutch trusted third party (Statistics Netherlands) used Record Identification Number pseudonymization to temporarily link health records on an individual level to facilitate analyses [23]. The presence of help-seeking was defined as the presence of a GP contact (either face-to-face, by phone or digital) for the reported symptom provided 6 weeks before, or 6 weeks after reporting a new-onset common somatic symptom during a Lifelines assessment. Presence of help-seeking within 3 months of symptom reported was defined using a similar strategy. Since we only included contacts with ICPC < 30 codes, we restricted our analyses to help-seeking for common somatic symptoms that the GP did not diagnose with an underlying disease (i.e. disease diagnosis; ICPC ≥ 70), but with a symptom diagnosis (i.e. symptoms that remain symptoms over time) [24].

### Statistical analyses

To assess whether sex and gender were independently associated with primary care help-seeking behavior, we applied generalized linear mixed-effect models with maximum-likelihood estimation. This approach allows for accounting for the dependency of residual errors due to the hierarchical structure of the data; these models also handle missing data efficiently. Our data were clustered on three levels: (1) observations of help-seeking (2) were nested within patients, (3) who in turn were nested in GP practices. Our initial model included only the intercept as independent variable and allowed intercepts to vary across individuals and GP practices (i.e. random intercepts for the second and third level). One-by-one we included independent variables as fixed effects. Thereafter, we also allowed the effects of sex and gender to vary across patients and GP practices (i.e. random intercepts and slopes for the second and third level). Model fit was assessed using the Akaike information criterion (AIC), with a lower AIC indicating better model fit. One-way ANOVAs were applied to assess the significance of differences in model fit. The random effects’ covariance matrix was unstructured and no mean centering of continuous independent variables was performed, as these had meaningful zero points.

Included independent variables were participants’ sex, age, educational level, score on the gender index, reported new-onset symptom, burden of somatic symptom experience at the moment of symptom reporting, and self-reported lifetime presence of chronic somatic and psychiatric diseases at the moment of symptom reporting. We also included a sex-by-gender interaction term to assess whether the association between gender and help-seeking differed for female and male participants. We repeated these analyses with help-seeking within 3 months as dependent variable.

In post hoc analyses, we assessed whether gender-related factors are associated with help-seeking. We repeated the abovementioned analyses, but replaced the gender index with gender-related factors, namely being a healthcare professional (yes/no), mean days per week one performs paid labor (1–7 days) and whether one considers oneself a homemaker (yes/no) as these were indicated by previous qualitative research to be of importance for help-seeking [9,13,14].

We assessed the presence of multicollinearity among independent variables, but found no indication of problems with multicollinearity as the variance inflation factor was ≤5 in all analyses [25]. We adhered to a two-sided *α*-level of 0.003, corrected for multiple comparisons (0.05/20; 19 independent variables and one interaction term within the family of tests). Data on independent variables were imputed as described earlier [6]. All analyses were conducted using the *lme4* package in R version 3.6.2 (R Foundation for Statistical Computing, Vienna, Austria).

## Results

### Patient population and GP consults

The Lifelines baseline population consisted of 152,728 adult participants, whereas the NPCD population comprised 277,881 patients. In total, we linked 20,187 individuals of whom 2709 (32.5%) male and 5616 (67.5%) female participants reported new-onset symptoms (Table 1).

**Table 1.** Characteristics of the study population at baseline (*N* = 8325).

		Male participants (*N* = 2709; 32.5%)	Female participants (*N* = 5616; 67.5%)
Age (years), mean (SD)		44.8 (12.7)	42.9 (13.0)
Gender index, median (IQR)		0.08 (0.02–0.30)	0.95 (0.82–0.99)
Educational level, *N* (%)	Low	905 (33.4%)	1724 (30.7%)
	Medium	968 (35.7%)	2229 (39.7%)
	High	836 (30.9%)	1663 (29.6%)
Symptom burden, mean SCL-90 SOM score (SD)		1.38 (0.37)	1.49 (0.41)
Chronic disease, *N* (%)	Present	1241 (45.8%)	3035 (54.0%)
	Absent	1468 (54.2%)	2581 (46.0%)
Self-rated health, *N* (%)	Good to excellent	2326 (87.1%)	4728 (85.4%)
	Poor to mediocre	345 (12.9%)	807 (14.6%)
New-onset symptom		5439	12,416
Help-seeking 6 weeks		70	185
Help-seeking 3 months		111	278

Ultimately, 255 individual participants (3.1%) sought help 360 times for 255 different symptoms within 6 weeks of reporting a new-onset symptom. Within 3 months of reporting a new-onset symptom, 387 individual participants (4.6%) sought help 596 times for 389 different new-onset symptoms (Table 2). The most frequently reported new-onset symptoms are muscle pains and (lower) back pain in 1037 (38.3%) and 903 (33.3%) of the male and in 1979 (35.2%) and 1626 (29.0%) of the female participants with new-onset symptoms, respectively (Table S3).

**Table 2.** Reported new-onset symptoms in Lifelines and concomitant GP contacts in 8325 participants.

New-onset common somatic symptom (ICPC)	Symptoms reported, *N* (%)	GP contacts within 6 weeks, *N* (%)	GP contacts within 3 months, *N* (%)
Headache (N01)	1868 (10.5%)	19 (7.5%)	26 (6.7%)
Dizziness (N17)	761 (4.3%)	17 (6.7%)	24 (6.2%)
Heart pain (K01)	483 (2.7%)	<10 (<3.9%)	<10 (2.6%)
(Lower) back pain (L02/L03)	2529 (14.2%)	88 (34.5%)	138 (35.5%)
Nausea (D09)	1406 (7.9%)	<10 (<3.9%)	<10 (<2.6%)
Muscle pain (L18)	3016 (16.9%)	20 (7.8%)	39 (10.0%)
Shortness of breath (R02)	633 (3.5%)	13 (5.1%)	17 (4.4%)
Hot-and-cold spells (A02)	1627 (9.1%)	<10 (<3.9%)	<10 (<2.6%)
Tingling extremities (N05)	1566 (8.7%)	10 (3.9%)	12 (3.1%)
Throat/swallowing problems (D21/R21)	798 (4.5%)	17 (6.7%)	25 (6.4%)
General tiredness (A04)	1698 (9.5%)	43 (16.9%)	66 (17.0%)
Arm/leg symptoms (L09/L14)	1470 (8.2%)	19 (7.5%)	28 (7.2%)
Total	17,855 (100.0%)	255 (100.0%)	389 (100.0%)

### Sex and gender in association with help-seeking behavior for somatic symptoms

We defined generalized linear mixed-effect models with increasing complexity to assess the associations between sex and gender, and help-seeking. For help-seeking within 6 weeks of new-onset symptom reporting, the model that allowed the effect of only sex (AIC = 2495.1), or of both sex and gender to vary across GP practices (AIC = 2501.1; i.e. models including random slopes) did not fit the data significantly better than the model that included only random intercepts for patients and GP practices (AIC = 2491.6; *χ*^2^_(DF=2)_ = 0.58, *p* = .75 and *χ*^2^_(DF=5)_ = 0.58, *p* = .99). Similarly, for the 3 month timeframe, the model that included random intercepts for patients and GP practices (AIC = 3454.4) fitted the data better than the models that allowed sex to vary across GP practices (AIC = 3458.1), or sex and gender to vary across GP practices (AIC = 3464.0). However, the differences in model fit were not statistically significant (*χ*^2^_(DF=2)_ = 0.37, *p* = .83 and *χ*^2^_(DF=5)_ = 0.41, *p* = .99, respectively). Therefore, we present the models including random intercepts, as these had the lowest AIC (Table 3).

**Table 3.** Generalized linear mixed-effects models: estimated associations between independent variables and help-seeking within different time frames.

		Help-seeking (*N* = 17,855)	
		Within 6 weeks	Within 3 months
Fixed effects		OR (95%CI)	
Female sex		**1.78 (1.13–2.80)**	**1.53 (1.05–2.21)**
Femininity		0.67 (0.39–1.16)	0.76 (0.49–1.20)
Age		**1.03 (1.02–1.04)**	**1.02 (1.01–1.03)**
Presence of chronic disease		1.00 (0.76-1.31)	1.04 (0.84–1.30)
Educational level	Low	Ref.	Ref.
	Medium	1.22 (0.89–1.67)	0.95 (0.74–1.23)
	High	1.18 (0.84–1.66)	0.94 (0.71–1.24)
Symptom burden		0.98 (0.96–1.01)	0.99 (0.97–1.01)
Presence of symptom	Headache	0.78 (0.41–1.48)	0.74 (0.43–1.28)
	Dizziness	1.77 (0.92–3.41)	1.67 (0.97–2.90)
	Heart pain	0.47 (0.14–1.58)	0.54 (0.21–1.38)
	(Lower) back pain	**2.67 (1.62–4.40)**	**3.00 (1.99–4.53)**
	Nausea	**0.29 (0.11–0.76)**	**0.31 (0.14–0.68)**
	Muscle pain	**0.48 (0.26–0.90)**	0.65 (0.40–1.06)
	Shortness of breath	1.57 (0.77–3.19)	1.40 (0.76–2.56)
	Hot-and-cold spells	**0.05 (0.01–0.32)**	**0.03 (0.01–0.22)**
	Tingling extremities	**0.47 (0.28–0.99)**	**0.38 (0.19–0.75)**
	Throat problems	1.67 (0.87–3.21)	1.68 (0.98–2.90)
	General tiredness	**2.10 (1.22–3.59)**	**2.24 (1.43–3.49)**
	Heavy arms/legs	1.27 (0.68–2.41)	1.33 (0.78–2.28)

The associations between the independent variables and seeking help within 6 weeks of symptom reporting are similar in effect size to those of help-seeking within 3 months of symptom reporting. Female patients seek help more often within 6 weeks of new-onset symptom reporting than male patients (OR = 1.78, 95%CI = 1.13–2.80), but femininity was not associated with help-seeking (OR = 0.67, 95%CI = 0.39–1.16). The sex-by-gender interaction terms were not statistically significant (OR = 0.54, 95%CI = 0.18–1.65), indicating that the association between gender and help-seeking within 6 weeks does not differ between female and male patients.

### Post hoc analyses

Femininity operationalized by the gender index showed no association with help-seeking for new-onset common somatic symptoms. We assessed whether specific gender-related factors that were identified by previous qualitative research were associated with help-seeking. We found that working as a healthcare professional or considering oneself as a homemaker showed no association with help-seeking within 6 weeks (OR = 0.93; 95%CI = 0.65–1.32 and OR = 0.68; 95%CI = 0.42–1.14, respectively). Weekly mean days of paid work did associate with help-seeking within 6 weeks (OR = 0.95; 95%CI = 0.91–0.98). No associations were found with help-seeking within 3 months.

## Discussion

To our knowledge, this is the first large-scale study that assesses separate associations of sex and gender with primary care help-seeking behavior for common somatic symptoms. Female sex was associated with help-seeking within 6 weeks and 3 months of symptom reporting, whereas femininity was not. We found increased mean working days per week to be negatively associated with help-seeking behavior.

### Strengths and limitations

This study had several strengths. First, we directly compared help-seeking in women and men, adjusted for the reported symptoms. Previous studies did not adjust for the reported symptoms and sex differences in presentation hereof. Second, previous studies assessing differences in primary care help-seeking behavior did not distinguish between sex and gender [11,26]. Here, we used a novel gender measure to disentangle associations of sex and gender with help-seeking behavior. Third, this study included consultation patterns based on primary care registries as opposed to self-reported measures, resulting in minimal risk of recall bias. Lastly, this study assessed help-seeking in the general population instead of clinical populations, which allowed for participants who have not sought help to be included in the analyses.

However, this study also had limitations. First, the NPCD is physician-centered and diagnosis-based, and the reason for encounter as reported by patients was not recorded. The use of recorded final diagnoses in this study implies that the frequency of help-seeking behavior was underestimated: GP consults in which a disease was recorded (i.e. ICPC ≥ 70) were excluded from analyses while those for which a symptom diagnosis was recorded were included (i.e. ICPC ≤ 30). This may explain the negative association between nausea, muscle pain, hot-and-cold spells and tingling extremities with GP contacts, as these are hardly diagnosed with symptom diagnoses. A recent study showed that men had a 6%-increase in odds of being provided with a disease diagnosis for their somatic symptoms compared to women [27]. This may imply that male help-seeking is underestimated in this study, and that the reported sex difference is overestimated.

Second, the exact moment of symptom onset is unknown and symptoms could have been present for a substantial amount of time before individuals decide to consult their GP. Possibly, patients already sought help for symptoms in an earlier stage which would erroneously classify these participants as non-help seekers. This may also lead to an underestimation of help-seeking behavior, potentially explaining why a mere 3.1% of the patients with new-onset symptoms sought help, which is less than reported by previous studies [28]. Moreover, many previous studies assessing primary care help-seeking behavior include symptoms beyond the 12 common somatic symptoms analyzed in this study, including sex-related symptoms and symptoms that require acute help-seeking, such as traumatic injuries, and do not distinguish between first and follow-up visits [2–4]. Although no studies about sex differences in the amount of time between the moment of first symptoms until the moment of first contact in primary care for common somatic symptoms are known to the authors, studies on symptoms associated with cancer and stroke among 10,297 and 162,856 adult patients, respectively, report no or inconclusive sex differences in time to help-seeking [29,30]. This suggests that no sex bias was introduced due to delays in help-seeking in this study.

### Comparison with previous studies

We found that female patients sought help more frequently from their GP for common somatic symptoms than male patients. This is in line with previous studies [1,2,8,31], including those that adjusted for sex-specific symptoms [5,15]. Other studies stated that the evidence for a sex difference in help-seeking for common somatic symptoms is weak [11,26]. However, these studies did not solely focus on primary care, included other symptoms than the current study, defined symptom experience very broadly, or relied on self-reported data about help-seeking behavior. These differences in methodology may result in differing outcomes between the studies.

Often, sex differences in help-seeking are, at least partly, attributed to gender differences between women and men [15]. However, the results of this study suggest that factors associated with sex differences in help-seeking should be sought in either the biological realm or in factors that go beyond the composite gender index. Multiple reasons grounded in biology have been explored for the female preponderance in help-seeking behavior. First, women experience, describe and report their symptoms in a different manner, and more readily attribute these to somatic causes than men [8,32]. Second, depressive and anxiety-related symptoms are more prevalent in women than in men, and these depressive and anxiety-related symptoms strongly associate with help-seeking [5,8]. Analyses in this study were adjusted for diagnosed psychiatric disorders, but not for depressive or anxiety-related symptoms. Last, female patients may have a lower threshold to seek help from their GP, as they are more familiar with primary care. This familiarity may arise, for example, because of more frequent visits to the GP related to gynecological interventions (i.e. the recurring smear test or pregnancy-related visits).

Gender-related factors that are not incorporated or are diluted in the gender index may also affect help-seeking. The gender index is based on psychosocial variables that predominantly reflect gender roles. Although studies show that more practical gender-related factors, such as household responsibilities [33,34], influence help-seeking behavior, others argue that mainly gender stereotypes influence people’s help-seeking behavior [35,36]. Gender stereotypes are not incorporated in the gender index, yet these do pose a social framework on people resulting in gendered behaviors and ideas. For example, traditional western gender stereotypes prescribe that it is more culturally and socially accepted for women to openly express their symptoms compared to men [6]. In contrast, these gender stereotypes state that men should be stoical about bodily experiences and should conceal their symptoms, even from care providers [36,37]. Although the gender index does not associate with help-seeking behavior, gender as an influencing factor on help-seeking should not be discarded completely.

### Clinical implications

This study found that female sex and the amount of days performing paid labor both associate with help-seeking behavior. For clinicians, the patient’s characteristics, such as sex, frequency of help-seeking and occupational factors, are pivotal in the clinical decision-making processes. Therefore, it is important for clinicians to be aware of female sex and gender-related factors being associated with primary care help-seeking [38]. Gender stereotyping, as mentioned above for example, may impose a social framework on people affecting help-seeking behavior. Awareness hereof may also counter patients’ delayed help-seeking behavior, which may result in delayed detection and concomitant treatment of symptoms.

Further research should consider whether the sex difference in help-seeking behavior also results in a sex difference in conducted diagnostic tests and diagnosed diseases [27,39]. Men are often typed as more reluctant seekers of healthcare and health information, whereas women are portrayed as frequent help-seekers. Such ideas may inadvertently prompt the GP to consider men’s symptoms as more serious than women’s symptoms, resulting in less watchful waiting in men.

## Supplementary Material

Supplemental Material

## Data Availability

Given the privacy of participants included in Lifelines and NPCD, the authors cannot publicly share the databases used in this study. Access to data can be requested by contacting Lifelines and NIVEL.
