# Supplementary material for: Sex and gender differences in primary care help-seeking for common somatic symptoms: a longitudinal study
Source: Scand J Prim Health Care. 2023 Mar 30;41(2):132–9. doi: 10.1080/02813432.2023.2191653 (PMC10193899; doi:10.1080/02813432.2023.2191653)
Supplement: Supplemental Material [file IPRI_A_2191653_SM1375.docx]

**Supplementary material to “****Sex and Gender Differences in Primary Care Help-seeking for Common Somatic Symptoms: a Longitudinal Study”**

Aranka V. Ballering, MSc*, Tim C. Olde Hartman, MD PhD, Robert Verheij, PhD, Judith G. M. Rosmalen, PhD

*a.v.ballering@umcg.nl

**Table S1: The SCL-90 SOM subscale**The Symptom CheckList-90 somatization subscale (SCL-90 SOM) is a subscale of the Symptom CheckList-90 (SCL-90). It asks participants to score their degree of bothering or distress due to twelve somatic symptoms in the past week. The total score ranges from 12–60, whereas the mean score of the SCL-90 SOM may range from 1-5.

| **How much in the past week were you bothered by:** | | **Not at all** | **A little bit** | **Moderately** | **Quite a bit** | **Extremely** |
| --- | --- | --- | --- | --- | --- | --- |
| **1** | Headache | 1 | 2 | 3 | 4 | 5 |
| **2** | Dizziness | 1 | 2 | 3 | 4 | 5 |
| **3** | Chest pain | 1 | 2 | 3 | 4 | 5 |
| **4** | Lower back pain | 1 | 2 | 3 | 4 | 5 |
| **5** | Nausea | 1 | 2 | 3 | 4 | 5 |
| **6** | Painful muscles | 1 | 2 | 3 | 4 | 5 |
| **7** | Difficulties breathing | 1 | 2 | 3 | 4 | 5 |
| **8** | Feeling hot and cold alternately | 1 | 2 | 3 | 4 | 5 |
| **9** | Numbness/tingling in parts of your body | 1 | 2 | 3 | 4 | 5 |
| **10** | Feeling a lump in your throat | 1 | 2 | 3 | 4 | 5 |
| **11** | Weakness in body parts | 1 | 2 | 3 | 4 | 5 |
| **12** | Heavy arms or legs | 1 | 2 | 3 | 4 | 5 |

**Table S2 B: Definition of new-onset common somatic symptoms**

New-onset symptoms were identified as symptoms that were not reported as present at baseline, but were reported as present during a follow-up measurement. Presence of symptoms was based on participants SCL-90 SOM score, as a score ≥3 (‘moderately’, ‘quite a bit’ and ‘extremely’) indicated presence of symptoms, whereas ≤2 (‘not at all’ and ‘a little bit’) indicated absence of symptoms.

| **Baseline** | **FUP1** | **FUP2** | **FUP3** | **New-onset symptom?** |
| --- | --- | --- | --- | --- |
| 0 | 0 | 0 | 0 | - |
| 0 | x | 0 | 0 | Yes, at FUP1 |
| 0 | x | x | 0 | Yes, at FUP1 |
| 0 | x | x | x | Yes, at FUP1 |
| 0 | x | 0 | x | Yes, at FUP1 |
| 0 | 0 | x | 0 | Yes, at FUP2 |
| 0 | 0 | x | x | Yes, at FUP2 |
| 0 | 0 | 0 | x | Yes, at FUP3 |
| x | 0 | 0 | 0 | - |
| x | x | 0 | 0 | - |
| x | x | x | 0 | - |
| x | x | x | x | - |
| x | 0 | x | 0 | - |
| x | 0 | x | x | - |
| x | 0 | 0 | x | - |
| x | x | 0 | x | - |

**Figure S1: Procedure and flowchart of included participants and GP consults**

We retrieved all GP consultations related to aforementioned common somatic symptoms from 2008 to 2018 in practices in the North of the Netherlands from the NPCD that could be linked to adult Lifelines participants. We assessed whether these consultations were associated with new-onset symptoms reported in Lifelines surveys. Consultations with the same ICPC codes as the aforementioned symptoms were regarded as related to the reported symptom. If participants contacted the GP multiple times within the assessed timeframe for the same symptom, we only included the first contacts.


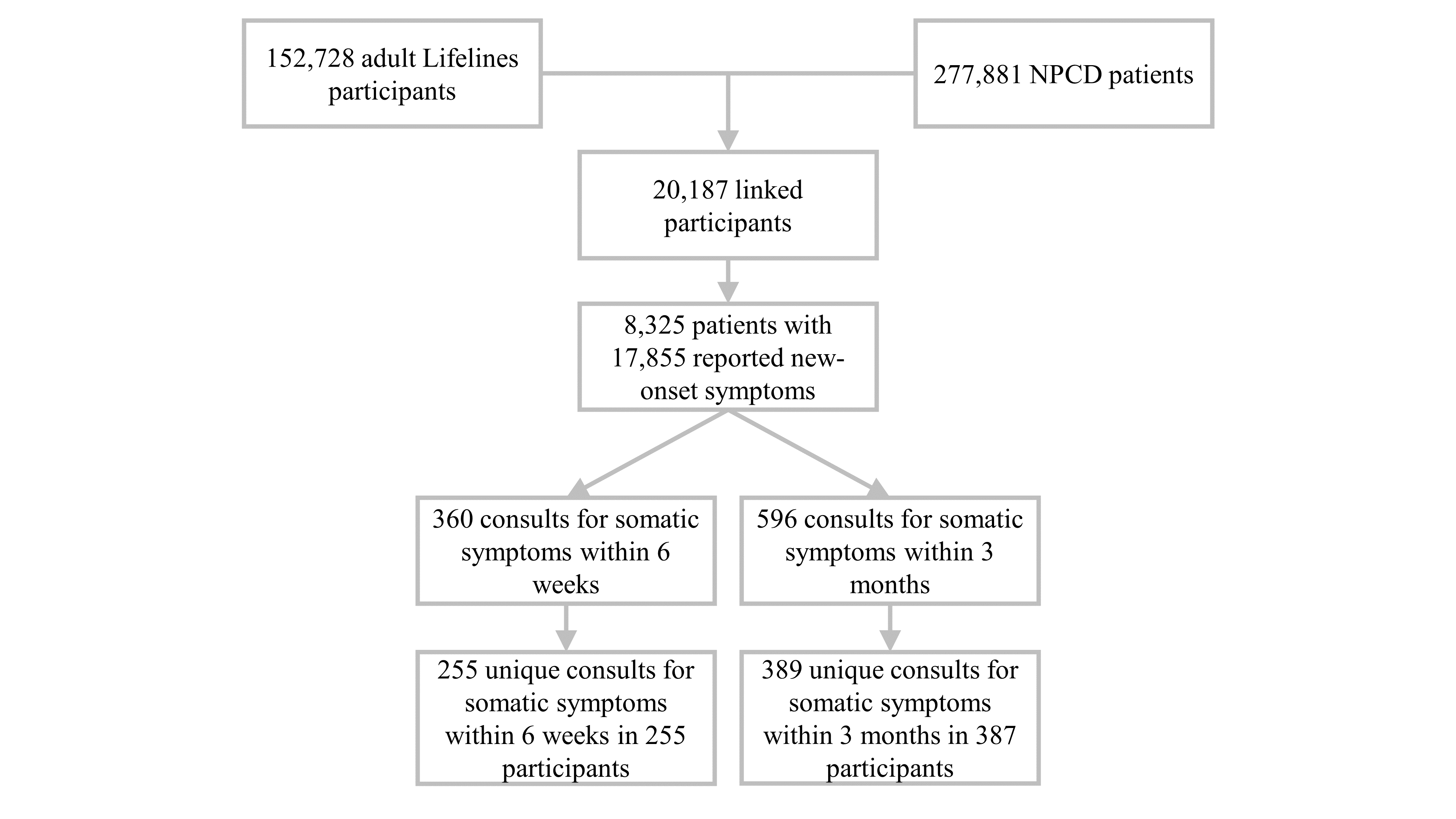


| **New-onset common somatic symptom (ICPC)** | **Reported symptoms, N (%)** | | **Help-seeking within 6 weeks, N (%)** | | **Help-seeking within 3 months, N (%)** | |
| --- | --- | --- | --- | --- | --- | --- |
|  | **Male (N=2,709)** | **Female (N=5,616)** | **Male (N=2,709)** | **Female (N=5,616)** | **Male (N=2,709)** | **Female (N=5,616)** |
| **Headache (N01)** | 477 (8.8%) | 1,391 (11.2%) | < 10 (<14.3%) | 16 (8.6%) | <10 (<9.0%) | 21 (7.6%) |
| **Dizziness (N17)** | 194 (3.6%) | 567 (4.6%) | < 10 (<14.3%) | 15 (8.1%) | <10 (<9.0%) | 21 (7.6%) |
| **Heartpain (K01)** | 175 (3.2%) | 308 (2.5%) | < 10 (<14.3%) | <10 (<5.4%) | <10 (<9.0%) | <10 (<3.6%) |
| **(Lower) backpain (L02/L03)** | 903 (16.6%) | 1,626 (13.1%) | 25 (35.7%) | 63 (34.1%) | 42 (37.8%) | 96 (34.5%) |
| **Nausea (D09)** | 376 (6.9%) | 1,030 (8.3%) | < 10 (<14.3%) | <10 (<5.4%) | <10 (<9.0%) | <10 (3.6%) |
| **Muscle pain (L18)** | 1,037 (19.1%) | 1,979 (15.9%) | < 10 (<14.3%) | 15 (8.1%) | 11 (9.9%) | 28 (10.1%) |
| **Shortness of breath (R02)** | 209 (3.8%) | 424 (3.4%) | < 10 (<14.3%) | <10 (<5.4%) | <10 (<9.0%) | 10 (3.6%) |
| **Hot-and-cold spells (A02)** | 289 (5.3%) | 1,338 (10.8%) | < 10 (<14.3%) | <10 (<5.4%) | <10 (<9.0%) | <10 (<3.6%) |
| **Tingling extremities (N05)** | 527 (9.7%) | 1,039 (8.4%) | < 10 (<14.3%) | <10 (<5.4%) | <10 (<9.0%) | 10 (3.6%) |
| **Throat/swallowing problems (D21/R21)** | 221 (4.1%) | 577 (4.6%) | < 10 (<14.3%) | 13 (7.0%) | <10 (<9.0%) | 18 (6.5%) |
| **General tiredness (A04)** | 571 (10.5%) | 1,127 (9.1%) | 16 (22.9%) | 27 (14.6%) | 23 (20.7%) | 45 (15.5%) |
| **Arm/leg symptoms (L09/L18)** | 460 (8.5%) | 1,010 (8.1%) | < 10 (<14.3%) | 15 (8.1%) | <10 (<9.0%) | 21 (7.6%) |
| **Total** | 5,439 (100.0%) | 12,416 (100.0%) | 70 (100.0%) | 185 (100.0%) | 111 (100.0%) | 278 (100.0%) |

**Table S3: Sex-stratified reporting of new-onset symptoms and help-seeking behavior**
